# Selections

**Published:** 1897-03

**Authors:** 


### Selections.

### OXIDATION.

A RECENT article by an eminent English physician calls attention in an especial manner to the prime necessity of oxidation in the economy of healthy humanity and causes us to realize, even more forcibly than we hitherto have done, how absolutely necessary oxidation is for health.

In the blood of an average human being there are continually circulating 2,250,000 of little red corpuscles, little bodies, little boats, so to speak, whose chief function is to receive a store of oxygen in the lungs, and carry it throughout the body.

When we realize that one can live for weeks without food, for days without water, but not for five minutes without air (the vitalizing ingredient of which is oxygen), are we not compelled to recognize the absolute necessity of oxygen?

When we remember that life is a constant molecular change, and when we realize that oxidation is the process of molecular change, are we not forced to rightly estimate the importance of oxygen to health?

Fresh air—ventilation—plenty of oxygen; they all mean the same thing, and they mean one of the greatest requisites of health.

### FORMALIN GELATIN: A NEW MODE OF ANTISEPTIC TREATMENT.

IN the *Therapeutische Monatschrift*, January, 1896, Dr. Schleich relates his experiences in the use of formalin gelatin in the treatment of wounds. The formalin gelatin is prepared by drying gelatin dissolved in water over formalin vapor. A firm, resistant, stony-hard, transparent body is thus formed. The question first to be decided was whether the gelatin would gradually dissolve and give off its formalin, and in this way set up a continued state of asepsis in its neighborhood. In the first experiment resection of intestine was performed on a rabbit, and before closing the abdominal wound a piece of formalin gelatin, the size of an apple, was introduced into the abdominal cavity. The animal was killed six and a half weeks later, and only a minute remnant of the gelatin was found in the midst of the newly-formed connective tissue. Further experiments were modified by the author to the extent that a quantity of virulent bacteria cultures was mixed with finely-powdered formalin gelatin and introduced into the system, all of which were absorbed without any reaction. These results led the author to use the gelatin in the treatment of wounds in the human subject. It was used in the form of powder, and Dr. Schleich became satisfied that it was gradually decomposed with continuous freeing of formalin, and consequent steady asepticism of the wound. Up to the time of writing he has used it in one hundred and twenty cases of acute suppuration, ninety-three aseptic healings of wounds, four compound fractures, and two deep scalp wounds, and he was in a position to state that by its means, all acute suppurations were cut short, and that in every wound an aseptic course could be guaranteed without the adoption of any further measures. Where necrotic tissue was present, however, it was powerless, as contact with sound tissue alone was able to set free the formalin. In order to render it serviceable in such cases a means must be discovered of setting the formalin free outside the body, and such a means has already been found by the author in a peptic acid solution (pepsin, 5 parts; hydrochloric acid, 0.3 part; distilled water to 100). The powder with which the wound is powdered requires moistening with the above pepsin solution. The mode of preparation of the formalin gelatin is given by the author.

The fact that when the gelatin was enclosed within the system it became eventually completely replaced by connective tissue led the author to still further experiments. These led to the conclusion that formalin gelatin, being procurable in any shape, and on being heated capable of being moulded to any form, it might be employed for the plastic connective tissue closure of defects of all kinds. Impregnated with lime salts, it proved itself capable of replacing pieces of bone removed in the course of resection.—*Berlin Cor. Med. Press and Circular.*

